# High Sensitivity SERS Substrate with Femtosecond Laser-Printed Nanohole Arrays

**DOI:** 10.3390/s25123680

**Published:** 2025-06-12

**Authors:** Yunfang Zhang, Dejun Liu, Han Liu, Yubin Deng, Zhiyong Bai, Changrui Liao, Yiping Wang, Ying Wang

**Affiliations:** 1 Shenzhen Key Laboratory of Photonic Devices and Sensing Systems for Internet of Things, Guangdong and Hong Kong Joint Research Centre for Optical Fibre Sensors, State Key Laboratory of Radio Frequency Heterogeneous Integration, Shenzhen University, Shenzhen 518060, China; zhangyunfang2017@email.szu.edu.cn (Y.Z.); 2200453023@email.szu.edu.cn (H.L.); 2210452004@email.szu.edu.cn (Y.D.); baizhiyong@szu.edu.cn (Z.B.); cliao@szu.edu.cn (C.L.); ypwang@szu.edu.cn (Y.W.); yingwang@szu.edu.cn (Y.W.); 2 Shenzhen Key Laboratory of Ultrafast Laser Micro/Nano Manufacturing, Key Laboratory of Optoelectronic Devices and Systems of Ministry of Education/Guangdong Province, College of Physics and Optoelectronic Engineering, Shenzhen University, Shenzhen 518060, China

**Keywords:** femtosecond laser-induced two-photon polymerization, nanohole arrays, SERS, rhodamine 6G

## Abstract

This article presents a novel method for fabricating repeatable and uniform surface-enhanced Raman scattering (SERS) substrates. The proposed method consists of two steps: (1) the fabrication of nanohole arrays using advanced femtosecond laser-induced two-photon polymerization (TPP) technology; and (2) the deposition of 9 nm thick silver nanoparticles on the nanohole arrays. The proposed nanohole arrays were optimized at the diameter, and the thickness of the silver film at two parameters. Regarding SERS substrates, a limit of detection of 10^−10^ M (rhodamine 6G) and analytical enhancement factors up to 3.5 × 10^4^ were achieved. At 1361 cm^−1^, the relative standard deviation (RSD) of the characteristic peak was 5.5%, demonstrating a highly reproducible SERS substrate.

## 1. Introduction

Over the past few decades, the deposition of metal nanoparticles and thin metal films onto precisely designed nanostructures has become an important research field that has promoted the development of catalysis, modern chemistry, biosensors, and medical diagnostics [1,2,3,4,5,6]. Nanomaterials based on nanoholes, created by depositing gold nanoparticles and thin metal films into hole structures smaller than the wavelength of the excitation laser, have shown potential in applications such as chemical and biological sensing, membrane biorecognition, and the study of adsorption characteristics of different amino acids and proteins [7,8,9,10]. The most attractive aspect of nanohole-based materials is their ability to generate unique optical responses under laser excitation based on changes in size and spacing, such as the surface plasmon resonance (SPR) of gold nanoparticles and localized surface plasmon resonance (LSPR) covering short-range ordered nanoholes [3,11]. These new types of nanomaterials have become the foundation of effective surface-enhanced Raman scattering (SERS) active substrates. This is because the typical SERS response of adsorbed analytes is highly sensitive to surface plasmon excitation, which can be easily provided by gold nanoparticles and gold nanoholes [3,12,13]. With the enhancement of the local electromagnetic field generated by SPR or LSPR, the SERS intensity of molecules near the nanohole surface also increases [14,15,16]. Therefore, it is desirable to deliberately control the size, shape, and spacing of gold nanoparticle arrays to produce LSPR wavelengths between the excitation wavelength and the molecular vibration band wavelength, thereby maximizing the enhancement of SERS signals [17]. Although SERS is considered a fast and highly sensitive technique for studying adsorbates on surfaces, and its sensitivity has been reported to be as low as the signal of a single rhodamine 6G molecule, its success largely depends on the performance of the SERS active substrate [18]. Developing a simple method to prepare ordered metal structures with reproducible size, spacing, and morphology for SERS active substrates is very necessary [19,20]. To date, template-assisted deposition, nanoimprint lithography, and electron beam lithography have been used to manufacture SERS active substrates, and their enhancement factors have been studied [12,13,21,22,23,24,25].

In this article, we propose a new method for the precise fabrication of reproducible surface-enhanced Raman scattering substrates based on femtosecond laser-induced two-photon polymerization technology. Using this advanced micro-nano processing technology, we successfully created nanohole arrays with specific hole sizes and deposited silver nanoparticles on these nanohole arrays via magnetron sputtering technology, thereby achieving high-performance SERS substrates. In this study, we particularly focused on the impact of two key parameters on the performance of SERS substrates: hole size and metal particle thickness. Experimental results indicate that the nanohole array with a 600 nm hole size demonstrated the best performance in Raman signal enhancement, with the SERS performance of the nanohole array improving as the hole size decreased, and that a 9 nm thick silver layer provided the strongest Raman spectral intensity. The optimized SERS substrate achieved a detection limit of 10^−10^ mol/L for rhodamine 6G, which is similar to the previous literature’s findings [26], and realized an enhancement factor of approximately 3.5 × 10^4^ that is consistent with reports in the literature [27]. This result indicates that the SERS substrates we prepared possessed high sensitivity and detection capabilities, which is of significant importance for biomedical detection and chemical-sensing fields. Furthermore, we explored the reproducibility of nanohole array SERS substrates. At 1361 cm^−1^, the relative standard deviation (RSD) of the characteristic peak was 5.5%, which is lower than the RSD of 7.1% from a plasmonic gold nanohole array substrate [26]. This high reproducibility is crucial for ensuring the reliability and stability of SERS substrates in practical applications.

## 2. Device Design and Fabrication

**Figure 1:**
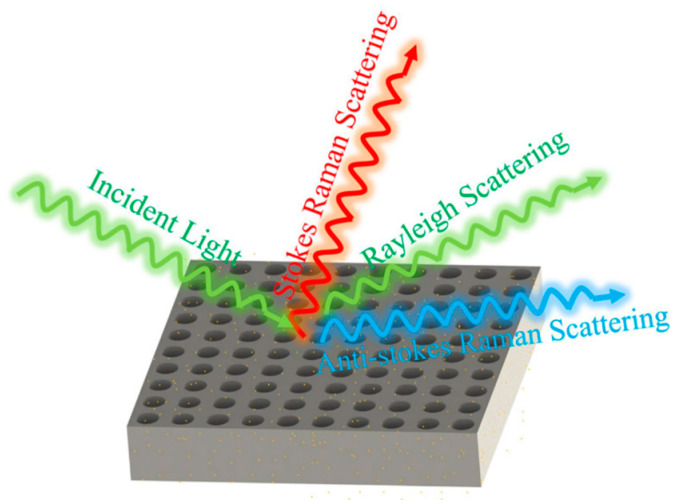
Schematic diagram of Raman scattering enhancement generated by nanohole arrays.

Figure 1 illustrates the structural schematic of a nanopore array surface-enhanced Raman scattering (SERS) substrate. This nanopore array was fabricated using femtosecond laser two-photon polymerization technology, which offers extremely high processing precision and resolution, enabling the creation of sub-micron level structures. The processing system employed in this study was the Photonic Professional GT2, produced by Nanoscribe in Germany. This advanced 3D laser direct writing system is widely used in the field of micro-nano fabrication. The system boasts extensive material compatibility and can process a variety of photosensitive materials. In this study, IP-Dip photoresist, which is compatible with a 63× objective lens, was selected. IP-Dip photoresist exhibits excellent optical and mechanical properties, allowing for high-precision three-dimensional structure fabrication under femtosecond laser irradiation, ensuring that the geometric shape of the nanopore array accurately replicates the design model.

The Photonic Professional GT2 system is capable of not only processing complex two-dimensional structures, but also fabricating arbitrary three-dimensional shapes. In this study, the geometric model of the nanopore array was designed and optimized using SolidWorks 2016 software. Upon completion of the design, the model file was directly imported into the Photonic Professional GT2 system for processing. The system converted the designed three-dimensional model into an actual nanostructure layer by layer through the femtosecond laser two-photon polymerization process, ultimately forming a nanopore array with high precision and consistency.

This processing method offers high flexibility and controllability, allowing for the adjustment of parameters, such as pore size and spacing of the nanopore array, according to experimental requirements, thereby optimizing the performance of the SERS substrate.

The fabrication process of the nanopore array surface-enhanced Raman scattering (SERS) substrate developed in this study consisted of three main steps.

First, the pre-designed three-dimensional structural model was imported into the control software of the Photonic Professional GT2 system. Femtosecond laser pulses with a central wavelength of 780 nm and a pulse duration of approximately 120 fs were focused onto the surface of a silica glass slide through a 63× objective lens with a numerical aperture (NA) of 1.40 and a working distance of 190 μm. This model, created using CAD software such as SolidWorks, precisely defined the geometric shape, pore size, spacing, and depth of the nanopore array. The system utilized femtosecond laser two-photon polymerization technology to focus the laser into the IP-Dip photoresist, scanning layer by layer according to the design model to form the outline of the nanopore array. After processing, the preliminary structure of the nanopore array was fixed on a glass substrate.

After processing, the sample underwent development and cleaning to remove uncured photoresist residues. First, the sample was immersed in a propylene glycol monomethyl ether acetate (PGMEA) solution for 20 min. Subsequently, the sample was transferred to isopropyl alcohol (IPA) for a 5 min immersion to further remove residual developer and adhesive. IPA, with its low surface tension, effectively cleaned the nanostructure surface, preventing structural collapse or damage. After cleaning, the sample was dried with nitrogen gas to ensure a clean and residue-free surface.

The developed and cleaned nanopore array structure required metal coating to impart surface-enhanced Raman scattering properties. The sample was placed in a magnetron sputtering coater, and a layer of metal nanoparticles (e.g., silver) was uniformly deposited on the surface of the nanopore array. The coating thickness was precisely controlled to ensure that the size and distribution of the metal particles effectively enhanced the Raman signal.

A Raman spectrometer (Alpha 300R, Witec, Ulm, Germany) with a confocal microscope was used for rhodamine 6G (R6G) detection, with a 532 nm frequency-stabilized single-mode diode laser included for Raman signal excitation. The sample was illuminated by a 532 nm laser beam with a power of 1 mW focused through a 100× microscopic objective (Olympus, Tokyo, Japan, 0.9 NA) during the test. For data collection, the integration time and scan number for averaging were 1 s and 10, respectively.

To optimize the surface-enhanced Raman scattering (SERS) signal intensity of the nanopore array, this study first systematically investigated the thickness of the silver (Ag) layer. Experiments were conducted on substrates with nanopore arrays featuring a pore diameter of 600 nm and a period of 900 nm. Using magnetron sputtering technology, silver layers with thicknesses of 5 nm, 7 nm, 9 nm, 10 nm, and 15 nm were deposited. By comparing the Raman signal intensities under different silver layer thicknesses, we aimed to determine the optimal silver thickness to achieve maximum SERS enhancement.

**Figure 2:**
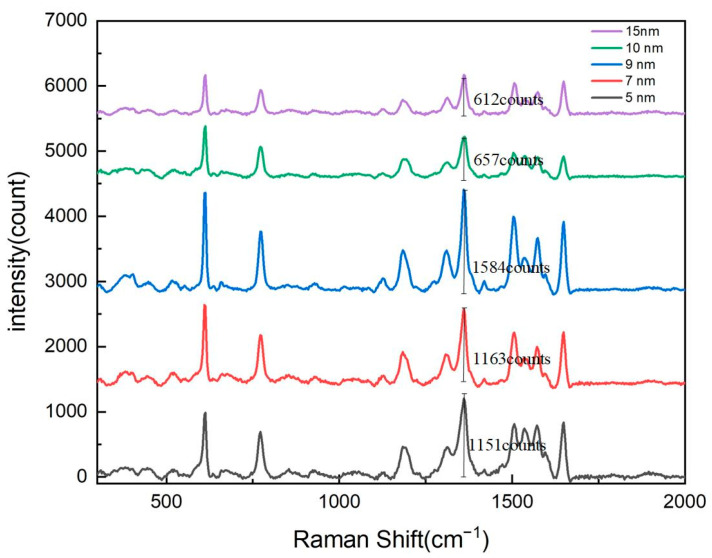
Raman spectroscopy response of nanohole arrays with varying silver film thicknesses.

The experimental results, as shown in Figure 2, demonstrate the significant influence of the silver layer’s thickness on the Raman signal’s intensity. Triplicate measurements were performed for each condition and were subsequently averaged. In the range of 5 nm to 9 nm, the Raman signal intensity of the nanopore array gradually increased with the thickness of the silver layer, reaching its maximum at 9 nm. This phenomenon can be attributed to the enhanced localized surface plasmon resonance (LSPR) effect caused by the increased silver thickness, which improved the local electromagnetic field enhancement. The LSPR effect is one of the primary mechanisms for SERS signal enhancement, and its intensity is closely related to the size, shape, and spacing of the metal nanostructures. When the silver layer’s thickness was moderate (e.g., 9 nm), the silver nanoparticles could effectively excite LSPR and form strong electromagnetic field hotspots on the surface of the nanopore array, significantly enhancing the Raman signals of adsorbed molecules.

However, when the silver layer’s thickness exceeded 9 nm, the Raman signal intensity began to gradually decrease. In the range of 9 nm to 15 nm, as the silver layer’s thickness further increased, the Raman signal intensity showed a declining trend. This phenomenon may have been caused by the following two reasons.

Weakened LSPR Effect: An excessively thick silver layer may have altered the size and spacing of silver nanoparticles, thereby weakening the LSPR effect. When the silver layer was too thick, the coupling between nanoparticles was reduced, leading to a decrease in the local electromagnetic field enhancement and a subsequent reduction in Raman signal intensity.

Limited Light Penetration and Molecular Adsorption: An overly thick silver layer may have restricted the penetration depth of the excitation light, reducing the interaction between light and adsorbed molecules. Additionally, a thick silver layer may have hindered the adsorption of molecules on the nanopore surface, further diminishing the Raman signal intensity.

**Figure 3:**
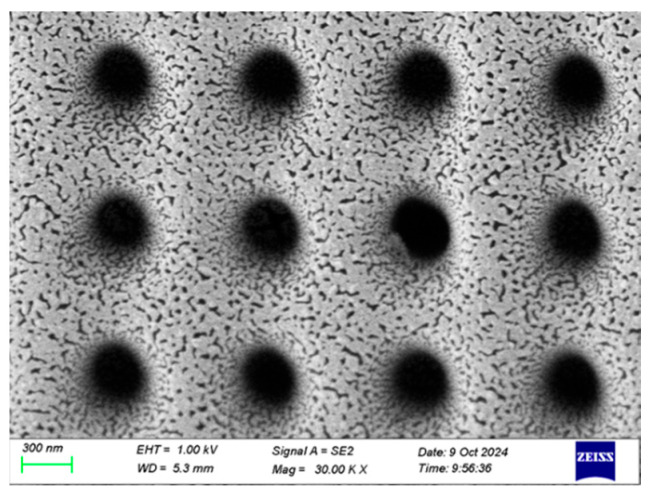
An SEM image of the sputtered 9 nm Ag layer.

Based on these experimental results, we concluded that the Raman signal intensity of the nanopore array reached its optimal value when the silver layer’s thickness was 9 nm. A magnified image of the 9 nm silver layer is shown in Figure 3. This finding is of great significance for the design and fabrication of high-performance SERS substrates. In practical applications, precise control of the silver layer’s thickness can maximize the performance of SERS substrates, thereby improving detection sensitivity and signal stability.

In addition to the silver layer’s thickness, the pore size of the nanopore array was also a critical factor influencing surface-enhanced Raman scattering (SERS) signal intensity. To investigate the effect of pore size on the Raman signal, this study designed and tested nanopore arrays with three different pore sizes: 600 nm, 700 nm, and 800 nm. Initially, we attempted to design nanopore arrays with smaller pore sizes; however, due to technical challenges during the development process, complete void structures could not be successfully formed for pores that were too small. Therefore, only nanopore arrays with pore sizes of 600 nm, 700 nm, and 800 nm were ultimately tested for Raman signal performance. In these experiments, the edge-to-edge distance between the pores was set to 300 nm, the silver layer’s thickness was optimized to 9 nm, and the concentration of the rhodamine 6G (R6G) used was 10^−5^ mol/L.

**Figure 4:**
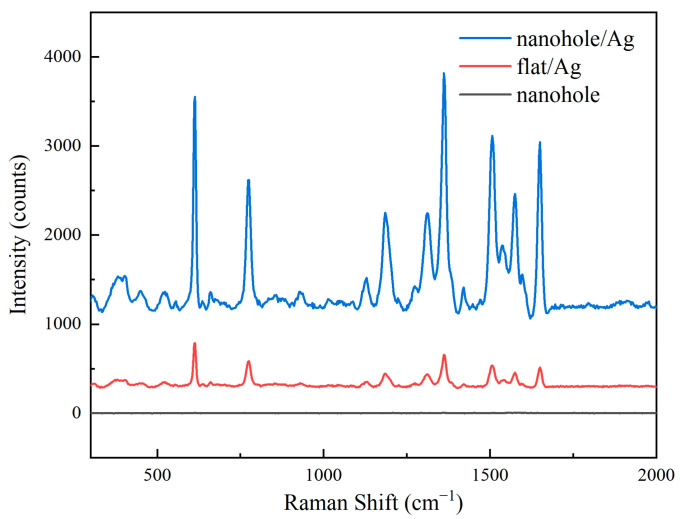
Raman spectroscopy of different substrates.

Figure 4 presents Raman spectra of three different substrates. These images show that the silver-coated nanohole array exhibited significantly enhanced Raman signals compared to the silver-coated planar substrate, while the uncoated nanohole array showed no discernible Raman signal.

**Figure 5:**
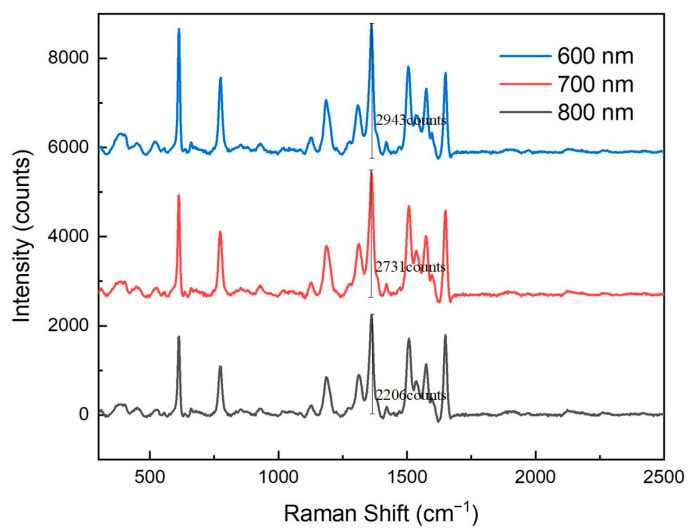
Raman spectroscopy response of nanohole arrays with different hole sizes.

Figure 5 presents Raman spectral responses of nanopore arrays with different pore sizes. It is evident from the figure that as the pore size of the nanopore array increased, the Raman signal intensity gradually decreased. Specifically, the 600 nm pore-size array exhibited the strongest Raman signal, while signal intensities of the 700 nm and 800 nm arrays decreased sequentially. This phenomenon indicates that smaller pore sizes are more conducive to the formation of intense electromagnetic field hotspots, thereby significantly enhancing the Raman signal.

The enhancement of the Raman signal primarily relies on the localized surface plasmon resonance (LSPR) effect. LSPR is a phenomenon that occurs when metal nanostructures are excited by light of a specific wavelength, greatly enhancing the local electric field and thus amplifying the Raman scattering signal of adsorbed molecules. When the pore size of the nanopore array was smaller, the spacing between the metal nanostructures decreased, leading to a more pronounced local electromagnetic field enhancement. Additionally, smaller pore sizes increased the specific surface area of the nanopore array, providing more adsorption sites, which further enhanced the Raman signal.

As the pore size increased, the spacing between the metal nanostructures also increased, resulting in a weakened LSPR effect. Larger pore sizes reduced the local electromagnetic field enhancement and decreased the number of adsorbed molecules, leading to a decline in Raman signal intensity. Therefore, the 600 nm pore-size nanopore array demonstrated the best Raman signal enhancement performance in these experiments.

## 3. Results and Discussion

Figure 4 presents scanning electron microscopy (SEM) images of nanopore arrays with different pore sizes. Each nanopore array had a size of 30 × 30 μm, with pore diameters of 600 nm, 700 nm, and 800 nm, respectively. The edge-to-edge distance between nanopores was uniformly set to 300 nm. The SEM images clearly show that the nanopore arrays were well ordered, with uniform pore sizes and highly consistent structural features.

**Figure 6:**
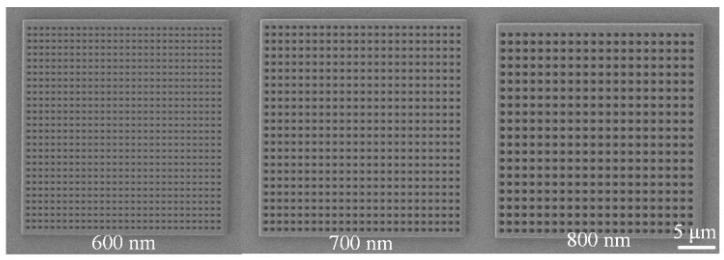
Scanning electron microscope (SEM) images of nanohole arrays with varying hole sizes.

The SEM images in Figure 6 clearly illustrate the structural characteristics of nanopore arrays with different pore sizes. The 600 nm, 700 nm, and 800 nm pore-sized arrays all exhibited highly ordered arrangements and uniform pore sizes, providing a reliable experimental basis for studying the influence of pore size on Raman signals. By optimizing the geometric parameters of the nanopore arrays, the LSPR effect and electromagnetic field enhancement can be further regulated, enabling the design and fabrication of high-performance SERS substrates.

**Figure 7:**
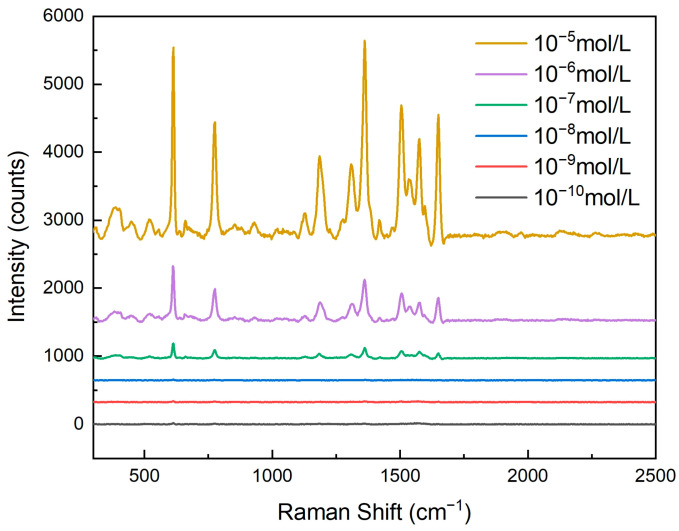
Raman spectroscopy response of nanohole arrays with a hole size of 600 nm and varying concentrations of rhodamine 6G.

As shown in Figure 7, by conducting Raman spectroscopy tests on rhodamine 6G (R6G) solutions of different concentrations, it could be observed that the activity of the nanopore array SERS substrate significantly increased with an increase in R6G concentration. This phenomenon was closely related to the adsorption behavior of R6G molecules on the surface of the nanopores. As the concentration of the R6G solution increased, more R6G molecules could adsorb onto the surface of the nanopore array, thereby enhancing the Raman signal intensity. Specifically, when the R6G concentration was low, the number of molecules adsorbed on the nanopore surface was limited, resulting in a relatively weak Raman signal. However, as the concentration gradually increased, more R6G molecules came into contact with the metal nanoparticles on the nanopores’ surface, creating more electromagnetic field hotspots and significantly enhancing the Raman signal.

For clear and convenient observation of the data, we measured the Raman spectral intensity at 611 cm^−1^, as shown in Table 1. The Raman intensity gradually increased with the rising concentration of R6G.

In this experiment, we tested R6G solutions with concentrations ranging from 10^−10^ mol/L to 10^−5^ mol/L. The results show that even at an extremely low concentration (10^−10^ mol/L), the nanopore array SERS substrate could still detect a Raman signal, indicating its exceptionally high sensitivity. This detection limit is primarily attributed to the optimized design of the nanopore array, including the pore size, the silver layer’s thickness, and the uniformity of the nanopore arrangement. These factors worked together to enable the substrate to generate a strong localized surface plasmon resonance (LSPR) effect, significantly enhancing the Raman signal of the adsorbed molecules.

The wavenumber and intensities of the major peaks were extracted from the baseline-corrected spectra and used in the following equation:

(1) $$\mathrm{AEF}=\frac{I_{\mathrm{SERS}}/C_{\mathrm{SERS}}}{I_{\mathrm{RS}}/C_{\mathrm{RS}}}$$

where I_SERS_ and C_SERS_ denote the peak intensity and concentration of the analyte for SERS measurements, and I_RS_ and C_RS_ represent the equivalent values for non-SERS measurements.

**Figure 8:**
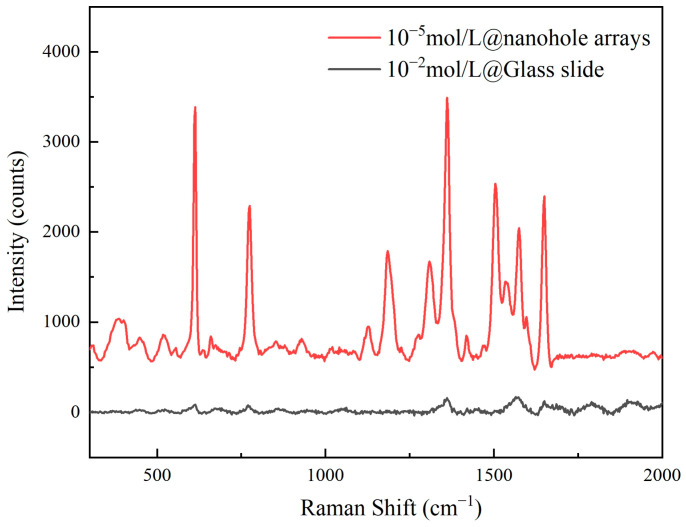
The Raman spectrum of the sample signal and reference spectrum.

When the rhodamine solution was at 10^−5^ mol/L, the enhancement factor of the nanoporous substrate reached 3.5 × 10^4^. Here, the reference spectrum was acquired at 10^−2^ mol/L concentration on a polished quartz slide. Figure 8 presents the comparative spectra between the two. For Raman signal measurements, identical parameters were employed: laser power at 1 mW, an integration time of 1 s, and 10 accumulations. The enhancement factor is one of the core parameters for evaluating the performance of SERS substrates, reflecting the substrate’s ability to amplify Raman signals. A high enhancement factor indicates that a substrate can significantly amplify Raman signals at the molecular level, enabling highly sensitive detection of low-concentration target molecules.

The magnitude of the enhancement factor primarily depends on the following factors.

Geometric parameters of the nanopore array: Parameters such as pore size, pore spacing, and pore depth directly influence the strength of the LSPR effect and the distribution of electromagnetic field hotspots.

Size and shape of metal nanoparticles: A silver layer thickness of 9 nm enables the optimal LSPR effect, maximizing the enhancement of the Raman signal.

Distribution of adsorbed molecules: Uniform adsorption of R6G molecules on the nanopore surface helps create more electromagnetic field hotspots, thereby increasing the enhancement factor.

In addition to enhancement effects, the uniformity and reproducibility of Raman signals from surface-enhanced Raman scattering (SERS) active substrates are also critical metrics for evaluating their performance. To more accurately assess the uniformity of Raman signal distribution on the nanopore array SERS substrate, we added 200 μL of a 10^−5^ M rhodamine 6G (R6G) solution to the substrate and allowed it to dry naturally at room temperature to ensure uniform adsorption of R6G molecules on the nanopore array surface. Subsequently, under consistent detection conditions, Raman spectra were collected from 10 random points on the substrate surface using a Raman spectrometer.

**Figure 9:**
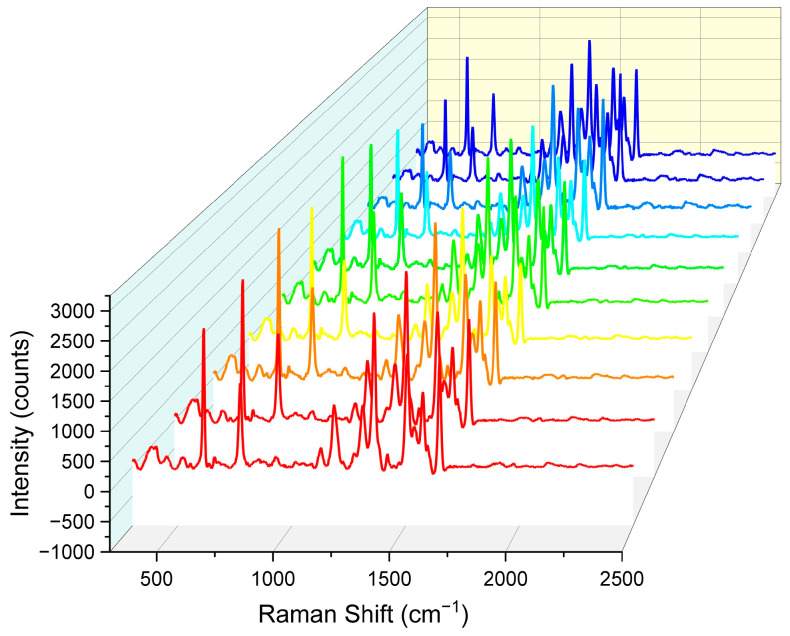
Comparison of Raman spectral intensity for 10 sets of nanohole arrays. Each color corresponds to the same Raman spectral parameters, with identical testing conditions.

As shown in Figure 9, SERS spectra from all 10 random points exhibited highly consistent trends and peak shapes. These 10 sets of data were from ten substrates processed with identical parameters. Specifically, the characteristic Raman peaks of R6G molecules were clearly visible in all spectra, with peak positions and relative intensities that were largely consistent. This result indicates that the nanopore array SERS substrate had excellent uniformity in Raman signal distribution, ensuring highly consistent Raman signals across different locations.

In addition to uniformity, we also verified the reproducibility of the nanopore array SERS substrate. Through multiple preparations and tests, it was found that the Raman signal intensity and characteristic peak positions remained consistent across different batches of substrates, demonstrating the substrate’s excellent reproducibility. This high reproducibility is primarily attributed to the precision and stability of the femtosecond laser two-photon polymerization technology and magnetron sputtering coating technology, which ensured consistent geometric parameters and metal nanoparticle distribution in each prepared substrate.

**Figure 10:**
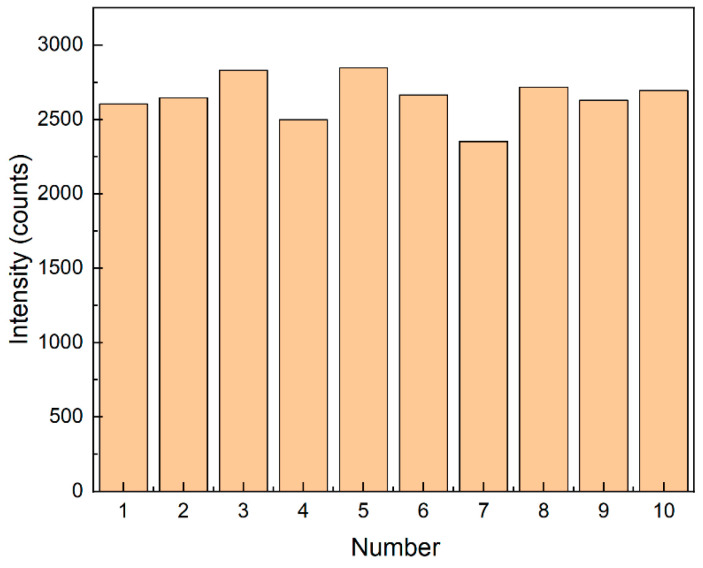
Bar chart of Raman spectral intensity at 1361 cm^−1^ for 10 groups.

The uniformity of spectral data reflects the distribution and performance of nanoparticles on the substrate. At the corresponding 1361 cm^−1^, the relative standard deviation (RSD) of the characteristic peak intensity was 5.5% (see Figure 10). The RSD was calculated using the following formula:

(2) RSD=SD/Mean×100%

(3) $$SD=\sigma =\sqrt{\frac{1}{N}\sum_{i=1}^{N}{\left(x_{i}-\mu \right)}^{2}}$$

In this formula, $x_{i}$ represents each data point, μ represents the mean value of the dataset, N is the total number of data points, and σ is the standard deviation.

## 4. Conclusions

In summary, this paper proposes a method for the precise fabrication of reproducible SERS substrates based on femtosecond laser-induced two-photon polymerization technology. The SERS substrates demonstrated were achieved by creating nanohole arrays through femtosecond laser-induced two-photon polymerization and subsequently depositing silver nanoparticles on the nanohole arrays via magnetron sputtering. This study optimized two parameters of nanohole SERS substrates. The first was the impact of hole size on the Raman signal, with a 600 nm hole size nanohole array showing better Raman enhancement effects. The other parameter was the impact of metal particle thickness on the Raman signal, with a 9 nm thick silver layer providing better Raman spectral intensity. The optimized nanohole array SERS substrate had a detection limit of 10^−10^ mol/L for rhodamine 6G testing and achieved an enhancement factor of approximately 3.5 × 10^4^. Additionally, this study explored the reproducibility of the nanohole array SERS substrate, with a highly reproducible SERS substrate showing a relative standard deviation of 5.5% at 1361 cm^−1^ for the characteristic peak. It is anticipated that the proposed SERS substrate will have advantages in cost-effective biomedical detection and chemical-sensing fields.

## Figures and Tables

**Table 1:** Intensities of Raman peaks of different R6G concentrations at 611 cm^−1^.

Concentrations (mol/L)	10^−10^	10^−9^	10^−8^	10^−7^	10^−6^	10^−5^
Intensities (counts)	12	15	18	184	779	2848

## Data Availability

Data are contained within the article.
